# An Analytical Study for (2 + 1)-Dimensional Schrödinger Equation

**DOI:** 10.1155/2014/438345

**Published:** 2014-01-27

**Authors:** Behzad Ghanbari

**Affiliations:** Department of Basic Sciences, Kermanshah University of Technology, Kermanshah, Iran

## Abstract

In this paper, the homotopy analysis method has been applied to solve (2 + 1)-dimensional Schrödinger equations. The validity of this method has successfully been accomplished by applying it to find the solution of some of its variety forms. The results obtained by homotopy analysis method have been compared with those of exact solutions. The main objective is to propose alternative methods of finding a solution, which do not require small parameters and avoid linearization and physically unrealistic assumptions. The results show that the solution of homotopy analysis method is in a good agreement with the exact solution.

## 1. Introduction

Stationary and time-dependent Schrödinger equation formulated by the Austrian physicist Erwin Schrödinger plays a fundamental role in physics for describing quantum mechanical [1].

In recent years, there exists a considerable number of works dealing with the problem of approximate solutions of the Schrödinger equation by using different methodologies, for example, Adomian decomposition method (ADM) [2], homotopy perturbation method (HPM) [3, 4], homotopy analysis method (HAM) [5], the boundary value method [6], and variational iteration method [7].

We consider the linear (2 + 1)-dimensional Schrödinger equation with variable coefficients of the form [6]

(1) −i∂u(x,y,t)∂t=∂2u(x,y,t)∂x2+∂2u(x,y,t)∂y2 +ω(x,y)u(x,y,t),u(x,y,0)=ϕ(x,y),

where *ω*(*x*, *y*) is an arbitrary potential function and $i=\sqrt{-1}$.

In this paper, an analytical method called the homotopy analysis method (HAM) will be used to solve (1).

The homotopy analysis method (HAM) was first proposed by Liao [8] in 1992, to get analytic approximations of highly nonlinear equations. Unlike other existing methods, this method is independent of small/large physical parameters, provides us a simple way to ensure the convergence of solution series, and gives us the great freedom to choose proper base functions.

These advantages make the method a powerful and flexible tool in mathematics and engineering, which can be readily distinguished from existing numerical and analytical methods [9, 10]. Recently, considerable research have been conducted in applying this method to a class of linear and nonlinear equations [10–15].

This paper is arranged in the following manner. In Section 2 the basic idea of standard HAM is illustrated. In Section 3, the implementation of this method on some examples is presented. Finally, conclusions are drawn in Section 4.

## 2. Standard Homotopy Analysis Method

Let us consider the differential equation

(2) $$\begin{array}{c} N\left[u\left(x,y,t\right)\right]=0, \end{array}$$

where *N* is a differential operator *x*, *y* and *t* denote independent variables, and *u*(*x*, *y*, *t*) is an unknown function.

Based on the constructed zero-order deformation equation by Liao [8], we give the following zero-order deformation equation in a similar way:

(3) $$\begin{array}{c} \left(1-p\right)L\left[\phi \left(x,y,t;p\right)-u_{0}\left(x,y,t\right)\right]=p\hbar N\left[U\left(x,y,t;p\right)\right], \end{array}$$

where *ℏ* ≠ 0 denotes an auxiliary parameter, *p* ∈ [0,1] is an embedding parameter, *L* is an auxiliary linear integer-order operator, *u*_0_(*x*, *y*, *t*) is an initial guess of unknown function *u*(*x*, *y*, *t*), and *ϕ*(*x*, *y*, *t*; *p*) is a kind of mapping, as described later. It is important that one has great freedom to choose auxiliary parameter *ℏ* in homotopy analysis method. If *p* = 0 and *p* = 1, it holds that

(4) $$\begin{array}{c} \phi \left(x,y,t;0\right)=u_{0}\left(x,y,t\right)=u\left(x,y,0\right),\\ \phi \left(x,y,t;1\right)=u\left(x,y,t\right). \end{array}$$

Thus as *p* increases from 0 to 1, the solution *ϕ*(*x*, *y*, *t*; *p*) varies from the initial guess *u*_0_(*x*, *y*, *t*) to the solution *u*(*x*, *y*, *t*). Expanding *ϕ*(*x*, *y*, *t*; *p*) in Taylor series with respect to *p*, one has

(5) $$\begin{array}{c} \phi \left(x,y,t;p\right)=u_{0}\left(x,y,t\right)+\sum_{m=0}^{\infty }u_{m}\left(x,y,t\right)p^{m}, \end{array}$$

where

(6) $$\begin{array}{c} u_{m}\left(x,y,t\right)=\frac{1}{m!}{\frac{\partial^{m}\phi }{\partial p^{m}}|}_{p=0}. \end{array}$$

If the auxiliary linear operator, the initial guess, and the auxiliary parameter *ℏ* are so properly chosen, the series (5) converges at *p* = 1, and one has

(7) $$\begin{array}{c} u\left(x,y,t\right)=u_{0}\left(x,y,t\right)+\sum_{m=0}^{\infty }u_{m}\left(x,y,t\right). \end{array}$$

According to the above, the governing equation can be deduced from the zero-order deformation, (3).

Define the vector

(8) $$\begin{array}{c} \vec{u}{}_{n}\left(x,y,t\right)=\left\{u_{0}\left(x,y,t\right),u_{1}\left(x,y,t\right),\ldots ,u_{n}\left(x,y,t\right)\right\}. \end{array}$$

Differentiation equation (3) *m* times with respect to the embedding parameter *p* and then setting *p* = 0 and finally dividing them by *m*!, we have the so-called *m*th-order deformation equation

(9) L[um(x,y,t)−χmum−1(x,y,t)]  =hRm−1[N[u→m−1(x,y,t)]],

where

(10) $$\begin{array}{c} R_{m}\left[N\left[\vec{u}{}_{m-1}\left(x,y,t\right)\right]\right]=\frac{1}{\left(m-1\right)!}{\frac{\partial^{m-1}N\left[\phi \left(x,y,t\right)\right]}{\partial p^{m-1}}|}_{p=0},\\ \chi_{m}=\{\begin{array}{cc} 1, & m>1,\\ 0, & m\leq 1. \end{array} \end{array}$$

The *m*th-order deformation equation (9) is linear and thus can be easily solved, especially by means of symbolic computation software such as Maple.

## 3. Application

To solve (1) by means of the standard HAM, we choose

(11) $$\begin{array}{c} L\left(u\right)=\frac{du}{dt}, \end{array}$$

(12) $$\begin{array}{c} N\left(\phi \right)=\frac{\partial \phi }{\partial t}-i\left[\frac{\partial^{2}\phi }{\partial x^{2}}+\frac{\partial^{2}\phi }{\partial y^{2}}+\omega \left(x,y\right)\phi \right]. \end{array}$$

According to (10)-(11) and (12), one has

(13) L[um(x,y,t)−χmum−1(x,y,t)]  =hRm−1[N[u→m−1(x,y,t)]],

where

(14) Rm[N[u→m−1(x,y,t)]]=∂um−1(x,y,t)∂t −i[∂2um−1(x,y,t)∂x2+∂2um−1(x,y,t)∂y2+ω(x,y)um−1(x,y,t)].

Substituting (11) and (14) into (13), we have

(15) um(x,y,t)=χmum−1(x,y,t) +h∫0tRm−1[N[u→m−1(x,y,ξ)]]dξ+cm,

where the constant of integration *c*_*m*_ is determined by the initial conditions

(16) $$\begin{array}{c} u_{m}\left(x,y,0\right)=0. \end{array}$$

The *m*th-order component *u*_*m*_(*x*, *y*, *t*)(*m* ≥ 1) would be achieved by means of symbolic computation software Maple, Mathematica, and so on.

We still have freedom to choose the auxiliary parameter *ℏ*. To investigate the influence of *ℏ* on the solution series, one can consider the convergence of approximation series related to a point in a domain [16]. These curves contain a horizontal line segment. This horizontal line segment denotes the valid region of *ℏ* which guaranteed the convergence of the related series.

## 4. Numerical Examples

We will illustrate the accuracy and efficiency of the homotopy analysis method applied to (1). For this purpose, here we present some numerical examples as considered in [4, 6].

**Example 1**

Consider (1) with *ω*(*x*, *y*) = 0 and the following initial condition:

(17) $$\begin{array}{c} u\left(x,y,0\right)=\sin\left(x\right)+\sin\left(y\right). \end{array}$$

The exact solution is given by

(18) $$\begin{array}{c} u\left(x,y,t\right)=e^{-it}\left(\sin\left(x\right)+\sin\left(y\right)\right). \end{array}$$

Starting with *u*_0_(*x*, *y*, *t*) = *u*(*x*, *y*, 0) and by using (15), we now successively obtain by HAM

(19) u1(x,y,t)=ℏit(sin⁡(x)+sin⁡(y)),u2(x,y,t)=[−12ℏ2t2+i(ℏ+ℏ2)t](sin⁡(x)+sin⁡(y)),u3(x,y,t)=[−ℏ2t2−ℏ3t2+i(ℏt+ℏ3t+2ℏ2t−16ℏ3t3)]×(sin⁡(x)+sin⁡(y)),u4(x,y,t)=[ℏ4t44!−3ℏ2t22−3ℏ4t22−3ℏ3t2+i×(ℏt+3ℏ2t−12ℏ3t3+3ℏ3t−12ℏ4t3+ℏ4t)]×(sin⁡(x)+sin⁡(y)),

and so forth.

**Figure 1:**
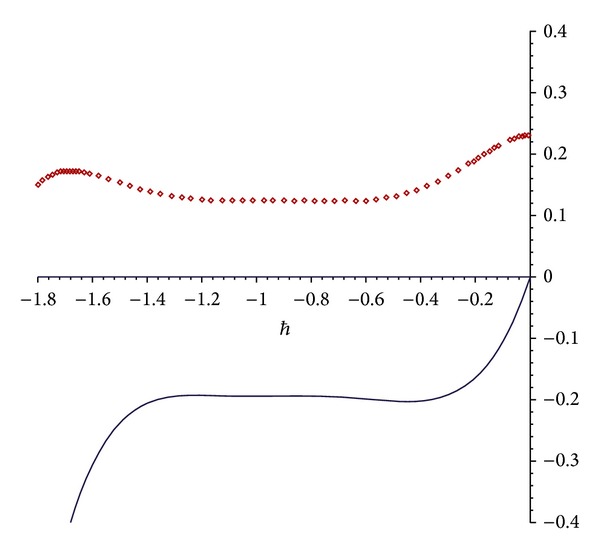
The *ℏ*-curves for the 7th-order of HAM approximation of *u*(0, *π*/2,0.5, *h*) for Example 1; dotted line: real part of approximation; solid line: imaginary part of approximation.

The proper value of *ℏ* = −1 is found from the *ℏ*-curve shown in Figure 1. Then the series solution expression is obtained by HAM as

(20) u(x,y,t)=[sin⁡(x)+sin⁡(y)] ×(1−it−12!t2+i3!t3+14!t4+⋯),

which clearly converges to the exact solution (18).

**Example 2**

Let us have *ω*(*x*, *y*) = 3 − 2tanh^2^(*x*) − 2tanh^2^(*y*) in (1) using the following initial condition:

(21) $$\begin{array}{c} u\left(x,y,0\right)=\frac{i}{\cosh\left(x\right)\cosh\left(y\right)}. \end{array}$$

The exact solution is

(22) $$\begin{array}{c} u\left(x,y,t\right)=\frac{ie^{it}}{\cosh\left(x\right)\cosh\left(y\right)}. \end{array}$$

Starting with *u*_0_(*x*, *y*, *t*) = *u*(*x*, *y*, 0) in recursive scheme (15), the following components are obtained:

(23) u1(x,y,t)=ℏt(1cosh⁡⁡(x)cosh⁡⁡(y)),u2(x,y,t)=[(ℏ+ℏ2)t−12ℏ2t2i](1cosh⁡⁡(x)cosh⁡⁡(y)),u3(x,y,t)=[2ℏ2t−16ℏ3t3+i(−ℏ2t2−ℏ3t2ℏt+ℏ3t)]×(1cosh⁡⁡(x)cosh⁡⁡(y)),u4(x,y,t)=[ℏt+3ℏ2t−12ℏ3t3+3ℏ3t−12ℏ4t3+ℏ4t+i(ℏ4t44!−3ℏ2t22−3ℏ4t22−3ℏ3t2)]×(1cosh⁡⁡(x)cosh⁡⁡(y)).

In this manner, the rest of components of the standard HAM solution can be found.

**Figure 2:**
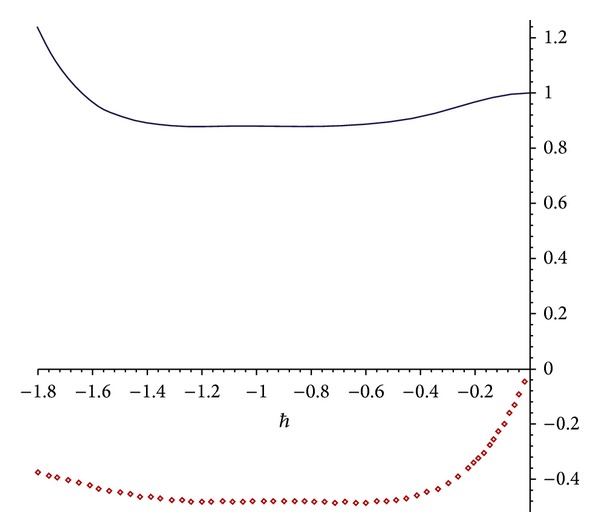
The *ℏ*-curves for the 7th-order of HAM approximation of *u*(0,0, 0.5, *h*) for Example 2; dotted line: real part of approximation; solid line: imaginary part of approximation.

Again, the value *ℏ* = −1 was chosen based on the *ℏ*-curve shown in Figure 2. Then the series solution expression is obtained by HAM as

(24) u(x,y,t)=icosh⁡⁡(x)cosh⁡⁡(y) ×(1−it−12!t2+i3!t3+14!t4+⋯),

which coincides with the exact solutions (22).

**Example 3**

As another example, let us consider (1) using *ω*(*x*, *y*) = 1 − 2/*x* ^2^ − 2/*y* ^2^, which has the following exact solution:

(25) $$\begin{array}{c} u\left(x,y,t\right)=x^{2}y^{2}e^{it}. \end{array}$$

We will solve this example directly by using HAM. We choose the initial approximation

(26) $$\begin{array}{c} u\left(x,y,0\right)=x^{2}y^{2}. \end{array}$$

Using such starting with *u*_0_(*x*, *y*, *t*) = *u*(*x*, *y*, 0), the following solutions are obtained:

(27) u1(x,y,t)=ℏtx2y2,u2(x,y,t)=[(ℏ+ℏ2)t−12ℏ2t2i]x2y2,u3(x,y,t)=[2ℏ2t−16ℏ3t3+i(−ℏ2t2−ℏ3t2ℏt+ℏ3t)]×ℏ2t2,u4(x,y,t)=[ℏt+3ℏ2t−12ℏ3t3+3ℏ3t−12ℏ4t3+ℏ4t+i(ℏ4t44!−3ℏ2t22−3ℏ4t22−3ℏ3t2)]×x2y2.

Plotting the *ℏ*-curve similar to what was plotted in Figure 2 suggests that we can take *ℏ* = −1. Then the series solution expression is obtained by HAM as

(28) $$\begin{array}{c} u\left(x,y,t\right)=x^{2}y^{2}\left(1+it-\frac{1}{2!}t^{2}-\frac{i}{3!}t^{3}+\frac{1}{4!}t^{4}+\cdots \right), \end{array}$$

which converges to the exact solution (25).

**Example 4**

As the last example, let us try to solve (1) with *ω*(*x*, *y*) = −4*x* ^2^ + 4*y* ^2^ − 4*x* − 4*y* − 1 and the following initial condition:

(29) $$\begin{array}{c} u\left(x,y,0\right)=e^{-({\left(x-1/2\right)}^{2}+{\left(y-1/2\right)}^{2})}. \end{array}$$

Its exact solution reads

(30) $$\begin{array}{c} u\left(x,y,t\right)=e^{-({\left(x-1/2\right)}^{2}+{\left(y-1/2\right)}^{2}+it)}. \end{array}$$

Starting with *u*_0_(*x*, *y*, *t*) = *u*(*x*, *y*, 0) in HAM procedure, we successively obtain

(31) u1(x,y,t)=−ite−((x−1/2)2+(y−1/2)2),u2(x,y,t)=[−12ℏ2t2+i(ℏ+ℏ2)t]e−((x−1/2)2+(y−1/2)2),u3(x,y,t)=[−ℏ2t2−ℏ3t2+i(ℏt+ℏ3t+2ℏ2t−16ℏ3t3)]×e−((x−1/2)2+(y−1/2)2),u4(x,y,t)=[ℏ4t44!−3ℏ2t22−3ℏ4t22−3ℏ3t2+i×(ℏt+3ℏ2t−12ℏ3t3+3ℏ3t−12ℏ4t3+ℏ4t)]×e−((x−1/2)2+(y−1/2)2),

and so forth. The value *ℏ* = −1 (which can be obtained by plotting the same *ℏ*-curve as was plotted in Figure 2) yields

(32) u(x,y,t)=e−((x−1/2)2+(y−1/2)2)×(1+it−12!t2+i3!t3+14!t4+⋯),

which converge to the exact solutions (30).

## 5. Conclusion 

In this paper, we have successfully developed homotopy analysis method to obtain the exact solutions of (2 + 1)-dimensional Schrödinger equation. It is apparently seen that these method are very powerful and efficient for solving different kinds of problems arising in various fields of science and engineering and present a rapid convergence for the solutions. Mohebbi and Dehghan in [6] reported the computed error for Examples 1–4, and in the present work we have obtained the exact solutions.
